# EnrichedHeatmap: an R/Bioconductor package for comprehensive visualization of genomic signal associations

**DOI:** 10.1186/s12864-018-4625-x

**Published:** 2018-04-04

**Authors:** Zuguang Gu, Roland Eils, Matthias Schlesner, Naveed Ishaque

**Affiliations:** 1 Division of Theoretical Bioinformatics (B080), German Cancer Research Center (DKFZ), Im Neuenheimer Feld 280, 69120 Heidelberg, Germany; 2 Heidelberg Center for Personalized Oncology (DKFZ-HIPO), German Cancer Research Center (DKFZ), Im Neuenheimer Feld 280, 69120 Heidelberg, Germany; 3 Department for Bioinformatics and Functional Genomics, Institute for Pharmacy and Molecular Biotechnology (IPMB) and BioQuant Center, Heidelberg University, Im Neuenheimer Feld 267, 69120 Heidelberg, Germany

**Keywords:** Visualization, Parallel heatmap, Genomic signal enrichment

## Abstract

**Background:**

High-throughput sequencing data are dramatically increasing in volume. Thus, there is urgent need for efficient tools to perform fast and integrative analysis of multiple data types. Enriched heatmap is a specific form of heatmap that visualizes how genomic signals are enriched over specific target regions. It is commonly used and efficient at revealing enrichment patterns especially for high dimensional genomic and epigenomic datasets.

**Results:**

We present a new R package named *EnrichedHeatmap* that efficiently visualizes genomic signal enrichment. It provides advanced solutions for normalizing genomic signals within target regions as well as offering highly customizable visualizations. The major advantage of *EnrichedHeatmap* is the ability to conveniently generate parallel heatmaps as well as complex annotations, which makes it easy to integrate and visualize comprehensive overviews of the patterns and associations within and between complex datasets.

**Conclusions:**

*EnrichedHeatmap* facilitates comprehensive understanding of high dimensional genomic and epigenomic data. The power of *EnrichedHeatmap* is demonstrated by visualization of the complex associations between DNA methylation, gene expression and various histone modifications.

## Background

With increasing accessibility and application of high throughput sequencing methods, there is a rise in the number of complex genomic and epigenomics studies. Thus, methods for integrative analysis are urgently required to provide comprehensive overviews of high dimensional multi-omics dataset to better understand biological systems [1]. Among them, effective visualization methods are of special importance as it helps to give an intuitive interpretation of the underlying data.

A common task for integrative visualization is to study how various genomic signals are enriched over specific genomic targets. Genomic signals can be represented as numeric values associating genomic locations, e.g. reads coverage in windows from whole genome sequencing data, DNA methylation rates for CpG sites from whole genome bisulfite sequencing data, or the intensities of histone modification in peak regions from ChIP sequencing data. The associated genomic signal values can also be binary to represent the existence of genomic features in the genome. While genomic targets are also genomic regions where the enrichment patterns are visualized. In many cases, genomic targets are gene-related features such as transcription start sites (TSS) or gene body. Generally, it can be any type of genomic features of interest, e.g. CpG islands (CGIs) if the aim is to study the methylation change at CGI borders. Current tools such as *deeptools* [2] and *ngs.plot* [3] are broadly used and successful at revealing potential enrichment patterns. However, they are limited at handling more complex cases without using external software, e.g. to summarize enrichment of signals difference of histone modifications between two subgroups of samples, or to visualize the correlation pattern between DNA methylation and expression of associated genes around TSS. Additionally, as stand-alone software tools, they are restricted to their built-in functionalities. For example, *deeptools* only supports to order rows by simple statistics such as row means in the heatmap, while it depends on external software to calculate more specific row orderings. *Genomation* [4] is an R package which visualizes enrichment for multiple types of signals simultaneously, but the functionality is very limited and difficult for more complex visualizations.

Here we present a new R package named *EnrichedHeatmap* that provides advanced and extensible solutions for summarizing and organizing enrichment heatmaps. Compared to available tools, the major advantages of *EnrichedHeatmap* are: 1) it is built on the framework of *ComplexHeatmap* package [5], thus enriched heatmaps can be flexibly combined with normal heatmaps and row annotation graphics, which makes it easy to integrate additional information to build complete overviews of the associations in complex datasets; 2) ordering and subgrouping rows in heatmaps are important for highlighting and comparing enrichment patterns. *EnrichedHeatmap* supports ordering methods such as pre-calculated orderings or flexible hierarchical clustering methods. *EnrichedHeatmap* also proposes new methods based on the closeness of signals regions relative to genomic targets to visualize how consistently close the signals are enriched to target regions. Also *EnrichedHeatmap* supports splitting of rows in heatmaps into groups by broad partitioning methods in R such as *k*-means or *k*-medoid clustering, or simply by a pre-defined category variable; 3) *EnrichedHeatmap* supports several methods to summarize mean signals for different types of genomic signals, depending on whether they are single point position-based signals or region-based signals. It also supports row smoothing to enhance the visual effect of the enrichment; 4) *EnrichedHeatmap* is capable of visualizing discrete signals such as chromatin state segmentations from *ChromHMM* [6]; 5) *EnrichedHeatmap* utilizes the *GRanges* data structure [7] which is the base data structure for handling genomic data in R and thus it can be seamlessly integrated into Bioconductor workflows; The power of *EnrichedHeatmap* is demonstrated by comprehensive visualization of various epigenomic signals over gene TSS to show the complex transcriptional regulation patterns.

## Implementation

Generally, the visualization of the signal enrichment over genomic targets can be standardized into two major steps where associations between genomic signals and target regions are firstly normalized into matrices and secondly the matrices are visualized as heatmaps with methods specifically for ordering rows to strengthen the pattern of enrichment. In this section, we describe the implementation of *EnrichedHeatmap* in detail and highlight the advantages and uniqueness of *EnrichedHeatmap* compared to other available tools.

### Normalize the associations

For a specific type of genomic signal (e.g. DNA methylation at CpG sites), associations to target regions are firstly normalized into a matrix where rows correspond to target regions e.g. gene-related regions and columns correspond to genomic windows around the targets. Target regions are extended upstream and/or downstream and the flanking regions are split into small windows of equal size. Each target is split into *k* windows as well with

$$ k=\left({n}_1+{n}_2\right)\bullet r/\left(1-r\right) $$

where *n*_1_ is the number of upstream windows, *n*_2_ is the number of downstream windows and *r* is the ratio of target columns presented in the matrix. Note, due to the unequal widths of target regions, widths of the windows inside targets are different for different targets as well. The default value of *r* is set as follows to ensures the mean width of target windows is the same as the width of upstream/downstream windows:

$$ r={\mu}_L/\left({\mu}_L+{L}_1+{L}_2\right) $$

where μ_L_ is the mean width of target regions. *L*_1_ and *L*_2_ are extensions of target regions in upstream and downstream.

**Fig. 1:**
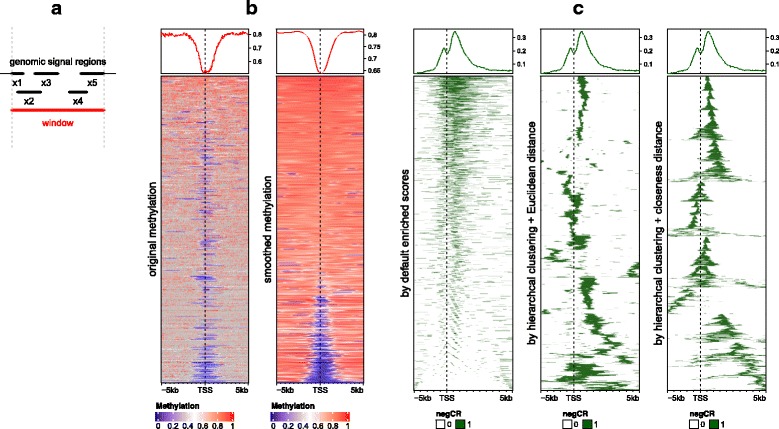
Implementation of EnrichedHeatmap. **a** Averaging model. The red line represents one window in the target regions or in the flanking regions when normalizing genomic signals to target regions. Black lines represent genomic signals that overlap to the given window. **b** Comparison between original methylation values and smoothed values. Grey color means no available methylation value associated for the window. Methylation data is from lung tissue in Roadmap dataset. Only data on chromosome 21 is used. Note the two heatmaps are independent and have different orderings. **c** Comparison between different row ordering methods. The three heatmaps correspond to ordering by enriched scores, by hierarchical clustering with Euclidean distance and by hierarchical clustering with closeness distance. The genomic signals are regions showing significant negative correlation between DNA methylation and expression of target genes

It is highly possible that multiple genomic signals overlap to one single window e.g. multiple CpG sites locating in one window, or one genomic signal spanning multiple windows. To summarize mean signal in every window, *EnrichedHeatmap* provides four averaging methods to summarize the signals for the window depending on whether the averaging is applied with background or not. As illustrated in Fig. 1a, for a given window (marked as red line), denote *n* as the number of signal regions which overlap to the window (it is 5 in Fig. 1a), *w*_*i*_ as the width of the intersected segment (black thick lines) for the *i*^th^ signal region, and *x*_*i*_ as the value associated with the signal region. If there is no value associated with the signal regions, *EnrichedHeatmap* sets *x*_*i*_ = 1 by default.

The “absolute” method denoted as *v*_*a*_ simply calculates the mean value from all signal regions regardless of their width:

$$ {v}_a=\frac{\sum_{i=1}^n{x}_i}{n} $$

The “weighted” method denoted as *v*_*w*_ calculates the mean value from all signal regions weighted by the width of their intersections:

$$ {v}_w=\frac{\sum_{i=1}^n{x}_i{w}_i}{\sum_{i=1}^n{w}_i} $$

“Absolute” and “weighted” methods are applied when background values should not be taken into consideration. For example, when summarizing mean DNA methylation in a window, non-CpG background should always be ignored, because methylation is only associated with CpG sites.

The “w0” method denote as *v*_*w*0_ calculates the weighted mean between the intersected segments and un-intersected parts:

$$ {v}_{w0}=\frac{\sum_{i=1}^n{x}_i{w}_i}{W+{W}^{\prime }} $$

where *W* is the sum of width of all intersected segments ($W={\sum}_{i=1}^n{w}_i$) and *W′* is the sum of width of the non-intersected parts. For example, the “w0” method can be applied to summarize mean histone modification intensity or mean CG content in a given window.

The “coverage” method denoted as *v*_*c*_ is defined as the mean signal averaged by the width of the window:

$$ {v}_c=\frac{\sum_i^n{x}_i{w}_i}{L} $$

where *L* is the width of the window itself. Note when *x*_*i*_ = 1, *v*_*c*_ is the mean base pair coverage for the signal regions overlapped in the window. Since signal regions may overlap to each other, thus *L* ≤ *W* + *W′*. When signal regions do not overlap to each other, “w0” method and “coverage” method are identical.

*EnrichedHeatmap* is capable of visualizing discrete signals. For a list of signals with *n* levels, internally *n* normalized matrices with “coverage” method are generated where each matrix corresponds to the enrichment of signal regions with one single signal level. When summarizing from *n* matrices into one final matrix, the signal levels are recoded with their numeric level orders, and for a single window, the numeric order of the signal level which shows maximum coverage is assigned to it. If none of the signal region overlaps to this window, zero value is assigned. *EnrichedHeatmap* has special visualization designed for discrete signal enrichment and since the final matrix is numeric, rows can be reordered by hierarchical clustering or partitioned by *k*-means clustering. Examples of visualizing discrete signals can be found in vignettes of the package.

*EnrichedHeatmap* supports smoothing of the average signals in the normalized matrix by local regression [8] or loess regression. It also imputes missing values by smoothing when no background value is provided. These functionalities particularly improve visualization for genomic signals that might be sparse in some parts of the genome, e.g. DNA methylation signals distal from CpG islands. On the other hand, a lot of other methods can be used to enhance *EnrichedHeatmap* only with a complete matrix without missing values, e.g. hierarchical clustering for row orderings. Figure 1b compares original methylation and smoothed methylation signals around gene TSS where rows are ordered by enriched scores (The definition of enriched scores will be introduced in a later section). It clearly shows smoothing dramatically improves the row ordering and the visual effect of the methylation heatmap. Since it can be possible that no CpG site exists in certain windows (window size is 50 bp in the two heatmaps in Fig. 1b) thus with no methylation values associated, it results in many grey grids in the first heatmap which represent missing values, which significantly disturbs the visualization. As a comparison, after smoothing and missing value imputation, it gives a clean and continuous methylation pattern in the heatmap. Although it might not be biologically correct to assign methylation values to non-CpG windows, it greatly improves the exploratory interpretability of the data.

**Fig. 2:**
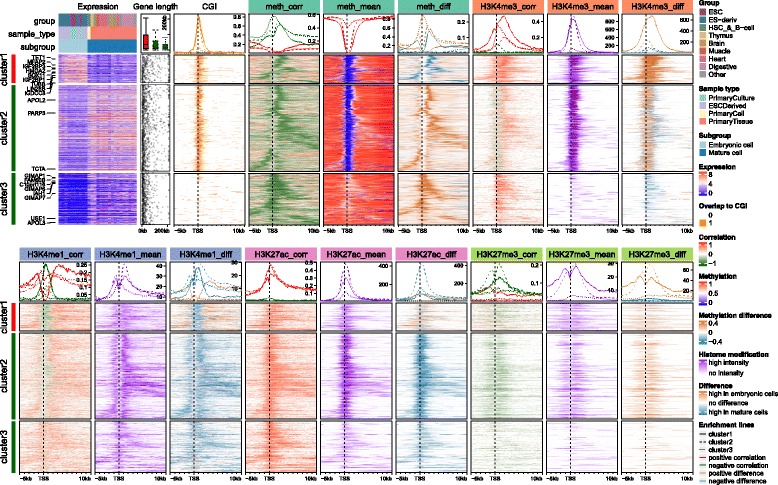
Comprehensive visualization of associations between gene expression, DNA methylation and four histone modifications from Roadmap dataset. In both top and bottom heatmap lists, rows correspond to same genes with different signals associated. Detailed explanation of data processing and R code for the plot can be found in Additional file 1

*EnrichedHeatmap* additionally supports a special scenario which associates signals to targets by mappings if the connections between signals and targets have already been constructed. By default, *EnrichedHeatmap* tries to overlap every signal region to every target region. However, there can be prior knowledge of the relations between signals and targets. In the example in Fig. 2 (this example will be discussed in detail in later section), we have defined a type of region named “correlated region” where it shows significant correlations between DNA methylation and expression of the host gene, in other words, there is already a gene associated to each correlated region. When normalizing correlated regions to gene TSS, it is possible that TSS of two genes are very close, and thus, correlated regions can be wrongly assigned to multiple genes if ignoring mappings between correlated regions and genes.

### Heatmap visualization

The normalized matrix is essentially a normal matrix with extra enrichment parameters attached. *EnrichedHeatmap* inherits and extends the *ComplexHeatmap* package, thus it provides great flexibility to arrange heatmaps as well as complex annotations, which is unique compared to other tools. On top of enriched heatmaps is a special type of annotation graphic which summarizes the enrichment across targets and can be directly corresponded to the patterns in the heatmap. An important feature of this annotation is it supports to summarize the positive and negative signals separately if signals to visualize are correlations or difference between subgroups (e.g. heatmap “meth_corr” in Fig. 2). Enrichment patterns are summarized separately if heatmaps are split by rows by *k*-means clustering or any pre-defined partitioning variables. With the framework of *ComplexHeatmap*, the enriched heatmaps can be concatenated with normal heatmaps as well as row annotations simply by “+” operator:

EnrichedHeatmap(…) + Heatmap(…) + rowAnnotation(…) + …

where the rows in all heatmaps and row annotations correspond and the main heatmap can be chosen to globally control the row ordering and subgrouping of all heatmaps.

Row ordering for the normalized matrix is crucial to enhance the patterns of enrichment. Rows can be ordered by certain types of scores calculated by rows (e.g. row means) or by clustering methods implemented in base or extended packages in R. *EnrichedHeatmap* provides two additional row ordering methods:

1. *Rows are ordered by enriched scores.* For each row in the normalized matrix, denote the vector for the associated values as ***x*** and it is split into ***x***_**1**_, ***x***_**2**_ and ***x***_**3**_ which correspond to values in upstream of the target, target itself and downstream of the target. The corresponding lengths of the three sub-vectors are denoted as *n*_1_, *n*_2_ and *n*_3_. The enriched score denoted as *s*_*e*_ is calculated as the sum of ***x*** weighted by the distance to target.

$$ {s}_e={\sum}_{i=1}^{n_1}{x}_{1i}\bullet \frac{i}{n_1}+{\sum}_{k=1}^{n_2}{x}_{2k}\bullet \left|{n}_2/2-\left|k-{n}_2/2\right|\right|+{\sum}_{j=1}^{n_3}{x}_{3j}\bullet \frac{n_3-j+1}{n_3} $$

Generally, when there is more signal centred on the target region, it has a higher enriched score.

2. *Rows are ordered by hierarchical clustering with closeness distance.* The column order in the normalized matrix represents the spatial order of windows located from upstream to downstream of the target. *EnrichedHeatmap* defines the closeness distance to measure how spatially close the signal regions of two different targets are based on the relative distance to targets. For any two rows in the normalized matrix where the associated values are denoted as ***x*** and ***y***, the distance based on closeness of signal regions in the two rows is defined as:

$$ {d}_{closeness}=\frac{\sum_{i=1}^n{\sum}_{j=1}^n\left|i-j\right|\bullet I\left(i,j\right)}{\sum_{i=1}^n{\sum}_{j=1}^nI\left(i,j\right)} $$

$$ I\left(i,j\right)=\left\{\begin{array}{c}1,\kern0.5em {x}_i\ne 0\ \mathrm{and}\ {y}_j\ne 0\\ {}0,\kern0.5em \mathrm{else}\end{array}\right. $$

Figure 1c compares row ordering by enriched scores, hierarchical clustering with Euclidean distance and hierarchical clustering with closeness distance. Note dendrograms generated by hierarchical clustering for rows in the latter two heatmaps are additionally reordered by the enriched scores to place enrichment patterns that are close to targets to the top of the heatmap as much as possible. Generally, when the top annotation which summarises mean enrichment across targets is added to the heatmap as well, ordering rows merely by enriched scores is not recommended because it provides redundant information as the top enriched annotation (left heatmap in Fig. 1c), and on the other hand, it fails to reveal spatial clusters as the other two methods. While hierarchal clustering with Euclidean distance is good at clustering enrichment patterns, it does not take column order into account, thus, it still can be possible that two spatially close clusters are separated in the heatmap (middle heatmap in Fig. 1c). By using closeness distance, it clearly sorts and clusters the enrichment patterns (right heatmap in Fig. 1c).

## Results

Figure 2 visualizes complex associations between gene expression, DNA methylation, and four histone modifications over gene TSS through a list of heatmaps by using Roadmap dataset [9]. In the analysis, 27 samples are separated into two subgroups that correspond to embryonic cells and mature cells. Rows are split according to differential expression and methylation pattern into three clusters. In each row cluster, rows are clustered based on the closeness of regions showing significant negative correlation between methylation and gene expression (we term them as “negCR”). For methylation and each histone modification, three heatmaps are used to illustrate the correlation to gene expression as well as the distribution of the signal among samples (by mean signals across all samples and mean signal differences between two subgroups). All heatmaps and annotations are arranged into two lines and rows in all heatmaps correspond to same genes. The top 10 most significantly differentially expressed genes between embryonic and mature cells are marked on left of the expression heatmap. A detailed explanation of data processing and step-by-step explanation of the R code can be found in Additional file 1.

Generally, genes in cluster 1 and 2 have high expression, long gene length (annotation “Gene length”) and low methylation over TSS (heatmap “meth_mean”) which correspond well with the enrichment of CpG islands over TSS (heatmap “CGI”), while genes in cluster 3 have low expression, short gene length, and intermediate mean methylation with almost no CGIs overlapping TSS. There is enrichment for significant negative CRs (negCRs) downstream of TSS in cluster 1 and 2 (solid and dashed green lines in annotation of “meth_corr” heatmap, the peaks of the enrichment locate at approximately + 2 kb of TSS.) while for cluster 3 genes, the enrichment of negCRs is very close to TSS. By associating the heatmap “CGI”, “meth_corr”, “meth_mean” and “meth_diff” together, we can make the conclusion that for genes in cluster 1 and 2, negCRs are enriched at the downstream border of CGI over TSS with high methylation variability, and even for cluster 3 genes there is also a trend that the negCRs are enriched at close downstream of TSS. This gives rise to the hypothesis that transcription factors can bind to chromatin in the gene body (in the lowly methylated negCRs) and are prevented to bind or move further into the gene body by DNA methylation after the negCRs.

H3K4me3 is a histone mark which is enriched at active TSS or promoters. Heatmap “H3K4me3_mean” shows strong enrichment of the mean signal over TSS for cluster 1 and cluster 2 genes with high expression. Such enrichment corresponds very well to the low TSS DNA-methylation. Interestingly, strong positive correlation to expression dominates in cluster 1 and the signals are significantly higher in embryonic cells (heatmap “H3K4me3_diff”). The peak for the enrichment of correlation signals in cluster 1 (solid red line in annotation of heatmap “H3K4me3_corr”) is broader than the mean signals while it is very similar as the enrichment peak for negCRs. For cluster 2 genes, the regions showing positive correlations are enriched at downstream border of H3K4me3 peaks while directly at the H3K4me3 peaks shows negative correlation although the correlation signals are weak and signal difference is small. Surprisingly, strong positive correlations dominate cluster 3 although the mean signals are very weak.

H3K4me1 is an active mark enriched at enhancers and promoter flanking regions. Nevertheless, it shows negative correlation at the TSS (solid and dashed green lines in annotation of heatmap “H3K4me1_corr”), especially strong for cluster 1. The peak for the negative correlation enrichment correlates well with CGI and low TSS-methylation, however the signals are low at TSS (heatmap “H3K4me1_mean”). Flanking TSS is dominated by positive correlations and the signal difference is comparably large in cluster 1 (solid brown line in annotation of heatmap “H3K4me1_diff”).

H3K27ac is also an active mark enriched in both active enhancers and promoters, and it generally shows positive correlations to expression in all three clusters (heatmap “H3K27ac_corr”). Interestingly the mean signals are the strongest in cluster 2 and mature cells have significantly higher signal intensity than embryonic cells (dashed blue line in annotation of heatmap “H3K27ac_diff”). The peak for the correlation signal enrichment is comparably broader than other marks.

H3K27me3 is a repressive mark and it generally shows negative correlation around TSS at relatively low level, excluding cluster 1 where there are no dominant correlation patterns (heatmap “H3K27me3_corr”). The signals are lower and sparser compared to other marks.

## Discussion

The heatmap visualization provides an intuitive way of showing the spatial associations between genomic signals and target regions. Here we have developed the *EnrichedHeatmap* package which facilitates the discovery of enrichment pattern of such associations. *EnrichedHeatmap* is capable of processing continuous signals, binary signals and discrete signals, and it provides different normalization methods for different types of genomic signals. More importantly, *EnrichedHeatmap* allows associating multiple sources of information through parallel heatmaps and annotations in an easy and modular way, which greatly facilitates the integrative analysis with multiple omic datasets.

The parallel heatmap visualization brings difficulty of setting proper row orders to discover patterns in all heatmaps simultaneously. Most of the available tools simply order rows based on the row means of the normalized matrix, which actually loses the information of how spatially similar the signal regions distribute in different target regions. Here we recommend ordering rows by hierarchical clustering on the normalized matrix as it highlights similar patterns for the signal regions that locate in spatially similar neighborhood of their associated target regions. Another difficulty raised is since there are multiple heatmaps that contain different data types, selecting a main heatmap to perform hierarchical clustering is also crucial for better displaying the association patterns. The solution to this problem depends on what key message users want to present. In Fig. 2, the hierarchical clustering is applied on the negCR matrix because the key message of the visualization is to show the association pattern between DNA methylation and gene expression around gene TSS. Moreover, since columns in the normalized matrix correspond to spatial distance to target regions, only clustering rows on subset of matrix which shows strong enrichment patterns helps to give a clearer view of the underlying pattern. E.g. in the vignette along with the package where the association between chromatin states and gene TSS is visualized, the row clustering is only applied to the subset of matrix which corresponds to 1 kb upstream and downstream of gene TSS because we observe there are very strong and consistent enrichment of active and bivalent TSS states in it while in flanking regions the chromatin states are more diverse and inconsistent.

Splitting rows in heatmaps helps to enhance the distinct patterns in different categories of target regions. *EnrichedHeatmap* allows splitting rows either by categorical variables or by *k*-means clustering. Generally speaking, the choice of how to split rows should be biological meaningful. In Fig. 2, rows of all heatmaps are split according to the methylation in 1 kb upstream and 2 kb downstream of gene TSS because we observe the methylation shows distinct difference and in the content of the analysis, methylation difference at gene TSS is always a dominant mark of transcription regulation.

## Conclusions

The *EnrichedHeatmap* package provides a flexible and powerful way to simultaneously visualize enrichment of various genomic signals over target regions. We believe it will be a useful tool for R/Bioconductor workflows to allow for more comprehensive understanding of high dimensional genomic and epigenomic data.

## Availability and requirements

**Project name:** *EnrichedHeatmap*

**Project home page:** http://bioconductor.org/packages/EnrichedHeatmap/, https://github.com/jokergoo/EnrichedHeatmap

**Operation systems:** Platform independent

**Programming language:** R (> = 3.3.0)

**License:** GPL (> = 2)

**Restrictions to use by non-academics:** None

## Additional files

Additional file 1: Data and source code for producing Figs. 1 and 2. (GZ 45195 kb)

## Abbreviations

CGI: CpG islands
CR: Correlated regions
negCR: Significantly negatively correlated regions
TSS: Transcription start sites
