# Supplementary material for: EnrichedHeatmap: an R/Bioconductor package for comprehensive visualization of genomic signal associations
Source: BMC Genomics. 2018 Apr 4;19:234. doi: 10.1186/s12864-018-4625-x (PMC5885322; doi:10.1186/s12864-018-4625-x)
Supplement: Supplementary file 1 — Data and source code for producing Figs. 1 and 2. (GZ 45195 kb) [file 12864_2018_4625_MOESM1_ESM.gz › EnrichedHeatmap-supplementary/supplS1.html]

Supplementary S1. Produce Figure 2 in the Manuscript


Table of Contents

- Supplementary S1. Produce Figure 2 in the Manuscript
  - General overview
  - Association between gene expression and methylation
  - Association with histone modifications
  - normalize CGI to gene TSS
  - Order and group genes
  - Organize heatmaps
  - Interpretation
  - Session Info

# Supplementary S1. Produce Figure 2 in the Manuscript

**Author**: Zuguang Gu ( z.gu@dkfz.de )

**Date**: 2018-02-02

---

To successfully run code in this supplementary, readers should install the newest version of following R packages.

```
library(devtools)
install_github("jokergoo/ComplexHeatmap")
install_github("jokergoo/EnrichedHeatmap")
```

Figure 2 in the manuscript visualizes comprehensive associations between various epigenomic signals from Roadmap
dataset. In this supplementary, we describe the methods and configurations used for visualizing the
associations.

The preprocessing of Roadmap data to fit into the analysis is relatively complex and is out of the scope of
this manuscript, thus here we only briefly describe the methods we use to process the data, while provide
more details on the visualization and interpretation part.

First load R packages and pre-calculated R objects which we will explain later.

```
library(EnrichedHeatmap)
library(GetoptLong)
library(circlize)
library(RColorBrewer)

load("roadmap_normalized_matrices.RData")
ls()
```

```
##  [1] "COLOR"              "expr"               "gene"               "gene_symbol"        "hist_mat_corr_list"
##  [6] "hist_mat_diff_list" "hist_mat_mean_list" "mat_cgi"            "mat_neg_cr"         "meth_mat_corr"     
## [11] "meth_mat_diff"      "meth_mat_mean"      "SAMPLE"             "tss"
```

## General overview

Roadmap dataset (http://egg2.wustl.edu/roadmap/web\_portal/) covers various human cell types and tissues and has been
uniformly processed. The dataset provides whole genome bisulfite sequencing data for DNA methylation, RNA sequencing
data for gene expression and ChIP sequencing data for various histone modifications.

As we observed, gene expression shows stronger correlation pattern with DNA methylation compared to other histone
modifications, thus, the whole integrative analysis is centered by gene expression and methylation, with associating
other histone modification signals, or in another words, we first look for regions where gene expression and methylation
is correlated, and as a second step we check how histone modification signals correlate or anti-correlate to these correlated
regions.

For the Roadmap dataset, we only use 27 samples which have both matched expression and methylation data with high
data quality and have consistent subgrouping in both expression and methylation datasets (results from an unpublished study). Here
`SAMPLE` contains annotations for samples under use and `COLOR` contains the corresponding colors for annotations.

```
SAMPLE
```

```
##        id        group    sample_type  subgroup
## E016 E016          ESC PrimaryCulture subgroup1
## E003 E003          ESC PrimaryCulture subgroup1
## E024 E024          ESC PrimaryCulture subgroup1
## E007 E007     ES-deriv     ESCDerived subgroup1
## E013 E013     ES-deriv     ESCDerived subgroup1
## E012 E012     ES-deriv     ESCDerived subgroup1
## E011 E011     ES-deriv     ESCDerived subgroup1
## E004 E004     ES-deriv     ESCDerived subgroup1
## E005 E005     ES-deriv     ESCDerived subgroup1
## E006 E006     ES-deriv     ESCDerived subgroup1
## E050 E050 HSC_&_B-cell    PrimaryCell subgroup2
## E112 E112       Thymus  PrimaryTissue subgroup2
## E071 E071        Brain  PrimaryTissue subgroup2
## E100 E100       Muscle  PrimaryTissue subgroup2
## E104 E104        Heart  PrimaryTissue subgroup2
## E095 E095        Heart  PrimaryTissue subgroup2
## E105 E105        Heart  PrimaryTissue subgroup2
## E065 E065        Heart  PrimaryTissue subgroup2
## E109 E109    Digestive  PrimaryTissue subgroup2
## E106 E106    Digestive  PrimaryTissue subgroup2
## E079 E079    Digestive  PrimaryTissue subgroup2
## E094 E094    Digestive  PrimaryTissue subgroup2
## E097 E097        Other  PrimaryTissue subgroup2
## E066 E066        Other  PrimaryTissue subgroup2
## E098 E098        Other  PrimaryTissue subgroup2
## E096 E096        Other  PrimaryTissue subgroup2
## E113 E113        Other  PrimaryTissue subgroup2
```

```
COLOR
```

```
## $group
##          ESC     ES-deriv HSC_&_B-cell       Thymus        Brain       Muscle        Heart    Digestive        Other 
##    "#924965"    "#4178AE"    "#678C69"    "#DAB92E"    "#C5912B"    "#C2655D"    "#D56F80"    "#C58DAA"    "#999999" 
## 
## $sample_type
## PrimaryCulture     ESCDerived    PrimaryCell  PrimaryTissue 
##      "#8cd2c7"      "#bfb9db"      "#faf7b4"      "#f57f73" 
## 
## $subgroup
## subgroup1 subgroup2 
## "#A6CEE3" "#1F78B4"
```

The 27 samples are separated into two subgroups (labeled as `subgroup` column in `SAMPLE`) where one subgroup
(`subgroup1`) corresponds to embryonic stem cells and the other subgroup (`subgroup2`) corresponds to primary tissues or
mature cells. Samples in the two subgroups show distinct difference in both expression and methylation datasets (results
from an unpublished study).

In this supplementary, we visualize the enrichment of various epigenomic signals around gene TSS with upstream 5kb and
downstream 10kb. All epigenomic signals have already been normalized to gene TSS and stored as R objects because the
calculation for the matrices involves complex data preprocessing and normalizing all datasets to gene TSS is time
comsuing. However, we will still provide pseudo code which generates these normalized matrices.

All epigenomic signals are normalized to gene TSS with same settings (upstream 5kb, downstream 10kb, window size 50bp),
thus, all normalized matrices have the same dimension and row order, and ith row, jth column in all matrices
correspond to a same position relative to a same gene.

There are following pre-calculated normalized matrices. Here we only simply describe these objects and details for generating
them will be explained in later sections.

- `mat_neg_cr`: a normalized matrix for correlated regions (CRs, The definition of CR is introduced in following
  sections) showing significant negative correlation between methylation and gene expression. The value in the matrix is
  whether a window is covered by negative CRs (values are 0 or 1).
- `meth_mat_corr`: a normalized matrix for all CRs. The value in the matrix is the mean correlation for CRs overlapped
  to a window.
- `meth_mat_mean`: a normalized matrix for mean methylation across all samples.
- `meth_mat_diff`: a normalized matrix for mean methylation difference between two subgroups.
- `mat_cgi`: a normalized matrix for CpG islands. The value in the matrix is whether a window is covered by CGIs.
- `hist_mat_corr_list`: a list of normalized matrices for the correlation between histone modification signals and gene
  expression. Each matrix corresponds to one type of histone modification.
- `hist_mat_mean_list`: a list of normalized matrices for the mean histone modification signals across all samples.
- `hist_mat_diff_list`: a list of normalized matrices for the histone modification signal difference between two
  subgroups.

Other R objects are

- `gene`: A `GRanges` object which contains positions of genes.
- `tss`: A `GRanges` object which contains positions of gene TSS.
- `gene_symbol` a mapping between Ensembl IDs and gene symbols.
- `expr`: gene expression matrix (Values are measured by `log2(RPKM + 1)`).

`tss` and `expr` also have the same row order as normalized matrices.

## Association between gene expression and methylation

When looking for associations between DNA methylation and gene expression, the process is gene centric. We briefly
describe the method as follows:

Each gene is extended to upstream 50kb and downstream 50kb to the full gene body. For each extended gene, we use a 6-CpG
sliding window with step of 3 CpGs and with maximum window size of 10kb. In each window, mean methylation is calculated
from the 6-CpG sites and the Spearman correlation as well as the correlaion test to expression of the current gene is
calculated. We term these 6 CpG windows as correlated regions (CRs) and significant CRs are filterred by FDR < 0.05
(from the correlation test) and methylation difference between the two subgroups are larger than 0.2.

According to this procedure, each CR belongs to one certain gene, which means, there is a mapping between CRs and genes.
Thus when normalizing CRs to TSS, this mapping should be provided so that CRs can be correctly normalized to their host genes.
Or else there can be scenarios that two extended genes overlap to each other and one CR of gene A also overlaps to gene B.
If the mapping is not provided, this CR will be wrongly mapped to gene B.

Assume `cr` contains CRs for all 6-CpG windows and the gene name column is named as `gene_id`, the correlation column
is named as `corr`, following code normalizes CRs to TSS.

```
# this chunk of code is only for demonstration
mat_corr = normalizeToMatrix(cr, tss, mapping_column = "gene_id", value_column = "corr", mean_mode = "absolute", ...)
```

Assume `sig_neg_cr` contains CRs showing significant negative correlations to expression, following code normalizes
significant negative CRs to TSS. Note there is no `value_column` in following code so that the normalized matrix
measures whether each window is covered by `sig_neg_cr`.

```
# this chunk of code is only for demonstration
mat_neg_cr = normalizeToMatrix(sig_neg_cr, tss, mapping_column = "gene_id", mean_mode = "absolute", ...)
```

The correlation itself does not tell the methylation level (highly methylated or lowly methylated) nor the variability
of methylation among samples. In order to get a more comprehensive view of the methylation, we also normalize mean
methylation and methylation variability to TSS. Methylation data represents as a matrix where rows are CpG sites and
columns are samples, thus we directly calculate mean methylation among samples with row means, then normalize the mean
methylation to gene TSS.

In following code, assume `meth` is a `GRanges` object of CpG sites and meta data columns contain methylation matrix for all samples.

```
# this chunk of code is only for demonstration
meth_mean = meth
mcols(meth_mean) = data.frame(mean_meth = rowMeans(mcols(meth)))
meth_mat_mean = normalizeToMatrix(meth_mean, tss, value_column = "mean_meth", mode = "absolute", ...)
```

And the mean methylation difference is calculated as \(m\_1 - m\_2\) where \(m\_1\) is the mean methylation matrix in subgroup
1 and \(m\_2\) is the mean methylation in subgroup 2.

```
# this chunk of code is only for demonstration
meth_mean_1 = meth
mcols(meth_mean_1) = data.frame(mean_meth = rowMeans(mcols(meth[, SAMPLE$subgroup == "subgroup1"])))
meth_mat_mean_1 = normalizeToMatrix(meth_mean_1, tss, value_column = "mean_meth", mode = "absolute", ...)
meth_mean_2 = meth
mcols(meth_mean_2) = data.frame(mean_meth = rowMeans(mcols(meth[, SAMPLE$subgroup == "subgroup2"])))
meth_mat_mean_2 = normalizeToMatrix(meth_mean_2, tss, value_column = "mean_meth", mode = "absolute", ...)
meth_mat_diff = meth_mat_mean_1 - meth_mat_mean_2
```

Since CRs are detected in upstream 50kb and downstream 50kb of the full gene, while for the visualizaiton, only upstream
5kb and downstream 10kb of TSS are used, genes which do not have a significant negative CR in [-5kb, 10kb] of TSS are
removed (can be filtered by `rowSums(mat_neg_cr) > 0`), which finnally results in 1832 genes for the analysis.

```
length(tss)
```

```
## [1] 1832
```

## Association with histone modifications

We use following four types of histone modifications which show specific patterns at gene TSS: H3K4me1, H3K4me3, H3K27ac
and H3K27me3. Similar as methylation, for each type of histone modification, there are three matrices which are 1.
correlation to gene expression; 2. mean signals across all samples; and 3. the mean signal difference between two
subgroups.

Being different from methylation datasets, the peak regions of histone modification
data across all samples cannot locate at a same genomic position which makes
it naturelly not a matrix-like data. To format it, we normalize signals to
gene TSS for eash sample separately, which generates a list of matrices with
same dimensions and settings.

Assume `peak` is a list of `GRanges` objects for peak regions in different samples.
In following, we additionally set `keep = c(0, 0.99)` to adjust outlier values which are
larger than 99th percentile.

```
# this chunk of code is only for demonstration
for(i in n_sample) {
    # assume the column name for the signals is called 'density'
    hm_list[[i]] = normalizeToMatrix(peak[[i]], tss, value_column = "density", keep = c(0, 0.99))
}
```

If we compress the list of matrices as a three-dimension array where the first dimension corresponds to genes,
the second dimension corresponds to windows and the third dimension corresponds to samples, the mean signal
across all sample can be calculated on the third dimension. Here `getSignalsFromList()` simplifies this job.

```
# this chunk of code is only for demonstration
hm_mat_mean = getSignalsFromList(hm_list, mean)
```

The mean difference between two subgroups can be calculated in a similar way:

```
# this chunk of code is only for demonstration
# hm_list_1 and hm_list_2 are normalized matrices for subgroup1 and subgroup2 separatedly
hm_mat_mean_1 = getSignalsFromList(hm_list_1, mean)
hm_mat_mean_2 = getSignalsFromList(hm_list_2, mean)
hm_mat_diff = hm_mat_mean_1 - hm_mat_mean_2
```

The correlation between histone modification and gene expression can
also be calculated on the third dimension of the array. In the user-defined function `fun`, `x` is the vector for gene i
and window j in the array, and `i` is the index of current gene.

```
# this chunk of code is only for demonstration
hm_corr = getSignalsFromList(hm_list, fun = function(x, i) {
    cor(x, expr[i, ], method = "spearman") # x = array[i, j, ]
})
```

We apply this method on all four types of histone modifications and normalized matrices are stored as `hist_mat_corr_list`,
`hist_mat_mean_list` and `hist_mat_diff_list`. All three objects are list of four matrices.

## normalize CGI to gene TSS

Normalizing CpG islands to TSS is straightforward. The value in the normalized matrix is whether each window is covered
by CpG islands.

```
# this chunk of code is only for demonstration
mat_cgi = normalizeToMatrix(cgi, tss, mean_mode = "absolute", ...)
```

## Order and group genes

The ordering of genes and sometimes separating genes into several groups are important to strenghen the effect of
visualization of the patterns. Especially when we have multiple heatmaps which share same row order, a proper way
to order genes is more important to highlight patterns for all heatmaps.

Which method to order genes depends on what pattern users want to reveal. In this analysis, the message we want to show
from the heatmaps are 1. the enrichment of significantly negatively correlated regions (negCRs) around TSS; 2. the difference between
subgroup 1 and subgroup 2; and 3. the different methylation patterns for different genes. The following procedures shows how we
group genes and how we order genes for better showing these patterns.

The expression difference between subgroup 1 and subgroup 2 samples is a major grouping factor. We construct a category
vector which corresponds to high expression and low expression in subgroup 1.

```
expr_mean = rowMeans(expr[, SAMPLE$subgroup == "subgroup1"]) - rowMeans(expr[, SAMPLE$subgroup == "subgroup2"])
expr_split = ifelse(expr_mean > 0, "high", "low")
expr_split = factor(expr_split, levels = c("high", "low"))
```

After looking at the methylation data, we found the methylation shows big difference between genes and the major
difference happens at small areas around TSS and flanks more to the downstream of TSS. Thus, we only extract 20% of
upstream of TSS and 40% of downstream of TSS and use methylation to separate genes into two subgroups which are low
TSS-methylation group and high TSS-methylation group.

The label of methylation groups are adjsuted that the first group always has lowest mean TSS-methylation.

```
set.seed(123)
upstream_index = length(attr(meth_mat_mean, "upstream_index"))
meth_split = kmeans(meth_mat_mean[, seq(round(upstream_index*0.8), round(upstream_index*1.4))], centers = 2)$cluster
x = tapply(rowMeans(meth_mat_mean[, seq(round(upstream_index*0.8), round(upstream_index*1.4))]), meth_split, mean)
od = structure(order(x), names = names(x))
meth_split = paste0("cluster", od[as.character(meth_split)])
```

Both grouping from expression and methylation is important, thus To make a more informative grouping of genes, the split
from expression and methylation are combined:

```
combined_split = paste(meth_split, expr_split, sep = "|")
```

There is one combined group with too few number of genes

```
tb = table(combined_split)
tb
```

```
## combined_split
## cluster1|high  cluster1|low cluster2|high  cluster2|low 
##           306           940            25           561
```

```
tb["cluster2|high"]/sum(tb)
```

```
## cluster2|high 
##    0.01364629
```

The proprotion of “cluster2|high” group is too small and also we don't want to make too many
row clusters, in order to make the plot more clear, “cluster2|high” is removed from the analysis.

Also all related variables should be subsetted to remove “cluster2|high” genes.

```
l = combined_split != "cluster2|high"
tss = tss[l]
expr = expr[l, ]
hist_mat_corr_list = lapply(hist_mat_corr_list, function(x) x[l, ])
hist_mat_mean_list = lapply(hist_mat_mean_list, function(x) x[l, ])
hist_mat_diff_list = lapply(hist_mat_diff_list, function(x) x[l, ])
mat_neg_cr = mat_neg_cr[l, ]
mat_cgi = mat_cgi[l, ]
meth_mat_corr = meth_mat_corr[l, ]
meth_mat_mean = meth_mat_mean[l, ]
meth_mat_diff = meth_mat_diff[l, ]
expr_split = expr_split[l]
meth_split = meth_split[l]
combined_split = combined_split[l]
n_row_cluster = length(unique(combined_split))
```

Next we calculate row order, following is a way which shows clear patterns for all signals.

From heatmaps which we show later, there is a clear pattern that the negCRs are enriched
at consistent positions downstream of TSS. To show this specific pattern, we designed a specific distance metric
which calculates how close the negCRs on two genes are based on relative distance
to TSS.

For two rows in the normalized matrix, assume \(a\_1, a\_2, …, a\_{n\_1}\) are the
window indices for one gene which overlaps with negative correlated
regions and \(b\_1, b\_2, … b\_{n\_2}\) are the indices for the other gene, the distance which is based
on closeness of the overlapped windows in the two genes is defined as:

\[ d\_{closeness} = \frac{\sum\_{i=1}^{n\_1} \sum\_{j=1}^{n\_2} {|a\_i - b\_j|} }{n\_1 \cdot n\_2}\]

Following code calculates row orders for genes. For each row cluster split by `combined_split`, rows are clustered
separately. In following code, `l_list` is a logical partition of genes and for each row cluster.

```
merge_row_order = function(l_list) {
    do.call("c", lapply(l_list, function(l) {
        if(sum(l) == 0) return(integer(0))
        if(sum(l) == 1) return(which(l))
        dend1 = as.dendrogram(hclust(dist_by_closeness(mat_neg_cr[l, ])))
        dend1 = reorder(dend1, -enriched_score(mat_neg_cr[l, ]))
        od = order.dendrogram(dend1)
        which(l)[od]
    }))
}

row_order = merge_row_order(list(
    combined_split == "cluster1|high",
    combined_split == "cluster1|low",
    combined_split == "cluster2|low"
))
```

## Organize heatmaps

After all matrics are generated, we can generate the complex heatmap list.

First we prepare the heatmap for expression. The columns of expression matrix
are only clustered for each subgroup.

```
dend1 = as.dendrogram(hclust(dist(t(expr[, SAMPLE$subgroup == "subgroup1"]))))
hc1 = as.hclust(reorder(dend1, colMeans(expr[, SAMPLE$subgroup == "subgroup1"])))
expr_col_od1 = hc1$order
dend2 = as.dendrogram(hclust(dist(t(expr[, SAMPLE$subgroup == "subgroup2"]))))
hc2 = as.hclust(reorder(dend2, colMeans(expr[, SAMPLE$subgroup == "subgroup2"])))
expr_col_od2 = hc2$order
expr_col_od = c(which(SAMPLE$subgroup == "subgroup1")[expr_col_od1], 
                which(SAMPLE$subgroup == "subgroup2")[expr_col_od2])
```

The first heatmap is the expression which is a normal heatmap where we put sample annotations on top.

```
ht_list = Heatmap(expr, name = "expr", show_row_names = FALSE,
    show_column_names = FALSE, width = unit(4, "cm"), show_column_dend = FALSE, 
    cluster_columns = FALSE, column_order = expr_col_od,
    top_annotation = HeatmapAnnotation(df = SAMPLE[, -1], col = COLOR, 
        show_annotation_name = TRUE, annotation_name_side = "left"),
    column_title = "Expression", column_title_gp = gpar(fontsize = 12),
    show_row_dend = FALSE, use_raster = TRUE, raster_quality = 2)
```

We extract top 20 genes with most significant p-values simply by t-test.

```
library(genefilter)
df = rowttests(expr, factor(SAMPLE$subgroup))
top_genes = rownames(df[order(df$p.value)[1:20], ])
```

These top genes are added as a text annotation.

```
index =  which(rownames(expr) %in% top_genes)
labels = gene_symbol[rownames(expr)[index]]
ht_list = rowAnnotation(sig_gene = row_anno_link(at = index, labels = labels,
        side = "left", labels_gp = gpar(fontsize = 10), link_width = unit(5, "mm"), 
        padding = 0.5, extend = unit(c(1, 0), "cm")), 
    width = max_text_width(labels, gp = gpar(fontsize = 10)) + unit(5, "mm")) + ht_list
```

On the right side of the expression is a row annotation which show the length of genes,
constructed by `rowAnnotation()` function and appended to the heatmap list.

```
gl = width(gene[names(tss)])
gl[gl > quantile(gl, 0.95)] = quantile(gl, 0.95)
ht_list = ht_list + rowAnnotation(gene_len = row_anno_points(gl, size = unit(1, "mm"), gp = gpar(col = "#00000040")), 
    width = unit(1.5, "cm"))
```

Add the heatmap which shows enrichment of CGI to TSS. Top annotation which shows enrichment pattern is added
by `anno_enriched()` function.

```
axis_name = c("-5kb", "TSS", "10kb")
ht_list = ht_list + EnrichedHeatmap(mat_cgi, col = c("white", "darkorange"), name = "CGI",
    column_title = "CGI", column_title_gp = gpar(fontsize = 12),
    top_annotation = HeatmapAnnotation(lines = anno_enriched(gp = gpar(col = "darkorange", 
        lty = 1:n_row_cluster), yaxis_facing = "left")), 
    axis_name = axis_name, axis_name_gp = gpar(fontsize = 8), use_raster = TRUE, raster_quality = 2)
```

Add the heatmap which shows the correlation between methylation and expression. Since in the normalized
matrix `meth_mat_corr`, there are positive correlations and negative correlations, both `pos_col` and `neg_col`
are set so that enrichment for positive correlation and negative correlation are drawn separately in the top annotation.

```
cor_col_fun = colorRamp2(c(-1, 0, 1), c("darkgreen", "white", "red"))
ht_list = ht_list + EnrichedHeatmap(meth_mat_corr, col = cor_col_fun, name = "meth_corr", 
    top_annotation = HeatmapAnnotation(lines = anno_enriched(gp = gpar(pos_col = "red", 
        neg_col = "darkgreen", lty = 1:n_row_cluster), yaxis_facing = "left")), 
    column_title = "meth_corr", column_title_gp = gpar(fontsize = 12),
    axis_name = axis_name, axis_name_gp = gpar(fontsize = 8), use_raster = TRUE, raster_quality = 2)
```

Add the heatmap which shows mean methylation among all samples.

```
meth_col_fun = colorRamp2(c(0, 0.5, 1), c("blue", "white", "red"))
ht_list = ht_list + EnrichedHeatmap(meth_mat_mean, col = meth_col_fun, name = "meth_mean", 
    column_title = "meth_mean", column_title_gp = gpar(fontsize = 12),
    top_annotation = HeatmapAnnotation(lines = anno_enriched(gp = gpar(col = "red", 
        lty = 1:n_row_cluster), yaxis_facing = "left")),
    , axis_name = axis_name, axis_name_gp = gpar(fontsize = 8), use_raster = TRUE, raster_quality = 2)
```

For the heatmap showing difference between subgroups, we define a function which generate color mappings showing
symmetric color mapping for positive difference and negative difference. This works for both
methylation difference and histone modification difference.

```
generate_diff_color_fun = function(x) {
    q = quantile(x, c(0.05, 0.95))
    max_q = max(abs(q))
    colorRamp2(c(-max_q, 0, max_q), c("#3794bf", "#FFFFFF", "#df8640"))
}

ht_list = ht_list + EnrichedHeatmap(meth_mat_diff, name = "meth_diff", col = generate_diff_color_fun(meth_mat_diff),
    column_title = "meth_diff", column_title_gp = gpar(fontsize = 12),
    top_annotation = HeatmapAnnotation(lines = anno_enriched(gp = gpar(pos_col = "#df8640", 
        neg_col = "#3794bf", lty = 1:n_row_cluster), yaxis_facing = "left")),
    axis_name = axis_name, axis_name_gp = gpar(fontsize = 8), use_raster = TRUE, raster_quality = 2)
```

Since here in the final heamtap list, there are 17 heatmaps which are too many to put in one row layout.
Thus, we separate the heatmaps into two and assign them to `ht_list_1` and `ht_list_2`. In following
code, the heatmaps for the last three histone modifications are assigned to the second heatmap list.

```
ht_list_2 = NULL
ht_list_1 = NULL
mark_name = names(hist_mat_corr_list)
for(i in seq_along(hist_mat_corr_list)) {
    # heatmaps for the 2nd, 3th and 4th histone modifications are assigned to a new `ht_list`
    if(i == 2) {
        ht_list_1 = ht_list
        ht_list = NULL
    }

    ht_list = ht_list + EnrichedHeatmap(hist_mat_corr_list[[i]], col = cor_col_fun, 
        name = qq("@{mark_name[i]}_corr"), column_title = qq("@{mark_name[i]}_corr"), 
        top_annotation = HeatmapAnnotation(lines = anno_enriched(gp = gpar(pos_col = "red",
            neg_col = "darkgreen", lty = 1:n_row_cluster), yaxis_facing = "left")), 
        top_annotation_height = unit(2, "cm"), 
        axis_name = axis_name, axis_name_gp = gpar(fontsize = 8), use_raster = TRUE, raster_quality = 2)

    ht_list = ht_list + EnrichedHeatmap(hist_mat_mean_list[[i]], 
        col = colorRamp2(c(0, quantile(hist_mat_mean_list[[i]], 0.95)), c("white", "purple")), 
        name = qq("@{mark_name[i]}_mean"), column_title = qq("@{mark_name[i]}_mean"), 
        column_title_gp = gpar(fontsize = 12),
        top_annotation = HeatmapAnnotation(lines = anno_enriched(gp = gpar(col = "purple", 
            lty = 1:n_row_cluster), yaxis_facing = "left")),
        axis_name = axis_name, axis_name_gp = gpar(fontsize = 8), use_raster = TRUE, raster_quality = 2)

    ht_list = ht_list + EnrichedHeatmap(hist_mat_diff_list[[i]], 
        col = generate_diff_color_fun(hist_mat_diff_list[[i]]), 
        name = qq("@{mark_name[i]}_diff"), column_title = qq("@{mark_name[i]}_diff"), 
        column_title_gp = gpar(fontsize = 12), 
        top_annotation = HeatmapAnnotation(lines = anno_enriched(gp = gpar(pos_col = "#df8640", 
            neg_col = "#3794bf", lty = 1:n_row_cluster), yaxis_facing = "left")),
        axis_name = axis_name, axis_name_gp = gpar(fontsize = 8), use_raster = TRUE, raster_quality = 2)

}
ht_list_2 = ht_list
```

We assign same `split` and `row_order` to both heatmap lists so that they can be correctedly corresponded.

All heatmaps are split by `combined_split`. Here we rename values in `combined_split` to `cluster1`, `cluster2` and `cluster3`.

```
split = as.vector(combined_split)
split[combined_split == "cluster1|high"] = "cluster1"
split[combined_split == "cluster1|low"] = "cluster2"
split[combined_split == "cluster2|low"] = "cluster3"
```

For each heatmap list, a single column heatmap (or bar) is attached to the most left side to represent difference
of expression between subgroups.

```
ht_list_1 = Heatmap(expr_split, show_row_names = FALSE, name = "expr_diff", 
    col = c("high" = "red", "low" = "darkgreen"), 
    show_column_names = FALSE, width = unit(2, "mm")) + ht_list_1
```

Now we explictly use `draw()` function to make the heatmaps because there are some global settings for all heatmaps.
We also decorate the final heatmap such as adding labels and axes.

```
ht_list_1 = draw(ht_list_1, 
    cluster_rows = FALSE, row_order = row_order, show_row_dend = FALSE,
    split = split, heatmap_legend_side = "bottom", gap = unit(2, "mm"))

add_boxplot_of_gene_length = function(ht_list) {

    row_order_list = row_order(ht_list)
    lt = lapply(row_order_list, function(ind) gl[ind])
    bx = boxplot(lt, plot = FALSE)$stats
    n = length(row_order_list)
    x_ind = (seq_len(n) - 0.5)/n
    w = 1/n*0.5
    decorate_annotation("gene_len", slice = 1, {
        rg = range(bx)
        rg[1] = rg[1] - (rg[2] - rg[1])*0.1
        rg[2] = rg[2] + (rg[2] - rg[1])*0.1
        pushViewport(viewport(y = unit(1, "npc") + unit(1, "mm"), just = "bottom", height = unit(2, "cm"), yscale = rg))
        grid.rect(gp = gpar(col = "black"))
        grid.segments(x_ind - w/2, bx[5, ], x_ind + w/2, bx[5, ], default.units = "native", gp = gpar(lty = 1:n))
        grid.segments(x_ind - w/2, bx[1, ], x_ind + w/2, bx[1, ], default.units = "native", gp = gpar(lty = 1:n))
        grid.segments(x_ind, bx[1, ], x_ind, bx[5, ], default.units = "native", gp = gpar(lty = 1:n))
        grid.rect(x_ind, colMeans(bx[c(4, 2), ]), width = w, height = bx[4, ] - bx[2, ], default.units = "native", 
            gp = gpar(fill = "white", lty = 1:n))
        grid.segments(x_ind - w/2, bx[3, ], x_ind + w/2, bx[3, ], default.units = "native", gp = gpar(lty = 1:n))
        grid.text("Gene length", y = unit(1, "npc") + unit(2.5, "mm"), gp = gpar(fontsize = 12), just = "bottom")
        grid.segments(unit(1, "npc") - unit(1, "mm"), c(0, 100000, 200000), unit(1, "npc"), c(0, 100000, 200000), default.units = "native")
    grid.text("200kb", unit(1, "npc") - unit(2, "mm"), unit(200000, "native") + unit(2, "mm"), default.units = "native", rot = 90, just = c("right", "bottom"), 
      gp = gpar(fontsize = 8))
    upViewport()
    })
}

add_boxplot_of_gene_length(ht_list_1)
# add background rectangles for column titles
i = 0
for(f in names(ht_list_1@ht_list)) {
    if(grepl("meth|H3K4me1|H3K4me3|H3K27ac|H3K27me3", f)) {
        decorate_column_title(f, {
            grid.rect(height = unit(0.8, "npc"), gp = gpar(fill = brewer.pal(8, "Set2")[as.integer(i/3)+1], col = NA))
            grid.text(ht_list_1@ht_list[[f]]@column_title, gp = gpar(fontsize = 12))
        })
        i = i + 1
    }
}
# axis for "Gene length" annotation
decorate_annotation("gene_len", slice = n_row_cluster, {
    grid.segments(c(0, 200000), unit(0, "npc"), c(0, 200000), unit(-1, "mm"), default.units = "native")
    grid.text("0kb", unit(0, "native") - unit(2, "mm"), unit(-2, "mm"), gp = gpar(fontsize = 8), just = c("left", "top"))
    grid.text("200kb", unit(200000, "native") + unit(1, "mm"), unit(-2, "mm"), gp = gpar(fontsize = 8), 
        just = c("right", "top"))
})
```

`split` and `row_order` are set to the second heatmap list as well.

```
ht_list_2 = Heatmap(expr_split, show_row_names = FALSE, name = "expr_diff", 
    col = c("high" = "red", "low" = "darkgreen"), 
    show_column_names = FALSE, width = unit(2, "mm")) + ht_list_2
ht_list_2 = draw(ht_list_2,
    cluster_rows = FALSE, row_order = row_order, show_row_dend = FALSE,
    split = split, heatmap_legend_side = "bottom", gap = unit(2, "mm"))
for(f in names(ht_list_2@ht_list)[-1]) {
    decorate_column_title(f, {
        grid.rect(height = unit(0.8, "npc"), gp = gpar(fill = brewer.pal(8, "Set2")[as.integer(i/3)+1], col = NA))
        grid.text(ht_list_2@ht_list[[f]]@column_title, gp = gpar(fontsize = 12))
    })
    i = i + 1
}
```

Note rows in the two heatmap list are all the same.

Following code only extracts the annotation graphics. We put all the annotation graphics in a layout so that
it is easily to compare between them.

```
add_anno_enriched = function(ht_list, name, ri, ci) {
    pushViewport(viewport(layout.pos.row = ri, layout.pos.col = ci))
    extract_anno_enriched(ht_list, name, newpage = FALSE)
    upViewport()
}

pushViewport(viewport(layout = grid.layout(nr = 3, nc = 6)))
add_anno_enriched(ht_list_1, "meth_corr",     1, 1)
add_anno_enriched(ht_list_1, "meth_mean",     1, 2)
add_anno_enriched(ht_list_1, "meth_diff",     1, 3)
add_anno_enriched(ht_list_1, "CGI",           1, 4)
add_anno_enriched(ht_list_1, "H3K4me3_corr",  2, 1)
add_anno_enriched(ht_list_1, "H3K4me3_mean",  2, 2)
add_anno_enriched(ht_list_1, "H3K4me3_diff",  2, 3)
add_anno_enriched(ht_list_2, "H3K4me1_corr",  2, 4)
add_anno_enriched(ht_list_2, "H3K4me1_mean",  2, 5)
add_anno_enriched(ht_list_2, "H3K4me1_diff",  2, 6)
add_anno_enriched(ht_list_2, "H3K27ac_corr",  3, 1)
add_anno_enriched(ht_list_2, "H3K27ac_mean",  3, 2)
add_anno_enriched(ht_list_2, "H3K27ac_diff",  3, 3)
add_anno_enriched(ht_list_2, "H3K27me3_corr", 3, 4)
add_anno_enriched(ht_list_2, "H3K27me3_mean", 3, 5)
add_anno_enriched(ht_list_2, "H3K27me3_diff", 3, 6)
upViewport()
```

## Interpretation

Figure 2 in the manuscript puts the two heatmap lists into one plotting page
and arranges legends to the right of the heatmaps (see this script).

Generally, genes in cluster 1 and 2 have high expression, long gene length
(annotation “Gene length”) and low methylation over TSS (heatmap “meth\_mean”)
which correspond well with the enrichment of CpG islands over TSS (heatmap
“CGI”), while genes in cluster 3 have low expression, short gene length, and
intermediate mean methylation with almost none CGIs overlapping TSS. There is
enrichment for significant negative CRs (negCRs) downstream of TSS in cluster 1 and cluster 2 (solid and dashed
green lines in annotation of “meth\_corr” heatmap, the peaks of the enrichment
locate at approximately +2kb of TSS.) while for cluster 3 genes, the
enrichment of negCRs is very close to TSS. By associating the heatmap “CGI”,
“meth\_corr”, “meth\_mean” and “meth\_diff” together, we can make the conclusion
that for genes in cluster 1 and cluster 2, negCRs are enriched at the downstream
border of CGI over TSS with high methylation variability, and even for cluster
3 genes, there is also a trend that the negCRs are enriched at close
downstream of TSS. It might give hypotheses that when the transcription
machine moves into the gene body from TSS, these exist some mechanism that
blocks this process and reflects on the changes of methylations.

H3K4me3 is a histone mark which is enriched at active TSS or promoters.
Heatmap “H3K4me3\_mean” shows strong enrichment of the mean signal over TSS for
cluster 1 and cluster 2 genes with high expression. Such enrichment corresponds very
well to the low TSS-methylation. Interestingly, strong positive correlation to
expression dominates in cluster 1 and the signals are significantly higher in
embryonic cells (heatmap “H3K4me3\_diff”). The peak for the enrichment of
correlation signals in cluster 1 (solid red line in annotation of heatmap
“H3K4me3\_corr”) is broader than the mean signals while it is very similar as
the enrichment peak for negCRs. For cluster 2 genes, the positive correlated
regions are enriched at downstream border of H3K4me3 peaks while directly at
the H3K4me3 peaks shows negative correlation although the correlation signals
are weak and signal difference is small. Surprisingly, strong positive
correlations dominate cluster 3 although the mean signals selves are very
weak.

H3K4me1 is an active mark enriched at enhancers and promoter flanking regions.
Nevertheless, it shows negative correlation at the TSS (solid and dashed green
lines in annotation of heatmap “H3K4me1\_corr”), especially strong for cluster
1. The peak for the negative correlation enrichment correlates well with CGI
and low TSS-methylation, however the signals selves are low at TSS (heatmap
“H3K4me1\_mean”). Flanking TSS is dominated by positive correlations and the
signal difference is comparably big in cluster 1 (solid brown line in
annotation of heatmap “H3K4me1\_diff”).

H3K27ac is also an active mark enriched in both active enhancers and
promoters, and it generally shows positive correlations to expression in all
three clusters (heatmap “H3K27ac\_corr”). Interestingly the mean signals are
the strongest in cluster 2 and mature cells have significantly higher signal
intensity than embryonic cells (dashed blue line in annotation of heatmap
“H3K27ac\_diff”). The peak for the correlation signal enrichment is comparably
broader than other marks.

H3K27me3 is a repressive mark and it generally shows negative correlation
around TSS at relatively low level, excluding cluster 1 where there are no
dominant correlation patterns (heatmap “H3K27me3\_corr”). The signals selves
are lower and sparser compared to other marks.

## Session Info

```
sessionInfo()
```

```
## R version 3.3.2 (2016-10-31)
## Platform: x86_64-apple-darwin13.4.0 (64-bit)
## Running under: macOS  10.13.2
## 
## locale:
## [1] en_GB.UTF-8/en_GB.UTF-8/en_GB.UTF-8/C/en_GB.UTF-8/en_GB.UTF-8
## 
## attached base packages:
##  [1] parallel  stats4    grid      methods   stats     graphics  grDevices utils     datasets  base     
## 
## other attached packages:
##  [1] genefilter_1.56.0     RColorBrewer_1.1-2    GetoptLong_0.1.8      EnrichedHeatmap_1.9.2 locfit_1.5-9.1       
##  [6] GenomicRanges_1.26.4  GenomeInfoDb_1.10.3   IRanges_2.8.2         S4Vectors_0.12.2      BiocGenerics_0.20.0  
## [11] ComplexHeatmap_1.17.1 circlize_0.4.3        knitr_1.18            markdown_0.8         
## 
## loaded via a namespace (and not attached):
##  [1] Rcpp_0.12.15         highr_0.6            pillar_1.1.0         XVector_0.14.1       bitops_1.0-6        
##  [6] tools_3.3.2          zlibbioc_1.20.0      digest_0.6.14        bit_1.1-12           memoise_1.1.0       
## [11] annotate_1.52.1      evaluate_0.10.1      RSQLite_2.0          tibble_1.4.2         lattice_0.20-35     
## [16] png_0.1-7            rlang_0.1.6          Matrix_1.2-12        DBI_0.7              stringr_1.2.0       
## [21] GlobalOptions_0.0.12 bit64_0.9-7          Biobase_2.34.0       AnnotationDbi_1.36.2 survival_2.41-3     
## [26] XML_3.98-1.9         blob_1.1.0           magrittr_1.5         splines_3.3.2        matrixStats_0.53.0  
## [31] shape_1.4.3          colorspace_1.3-2     xtable_1.8-2         stringi_1.1.6        RCurl_1.95-4.10     
## [36] rjson_0.2.15
```
