# Supplementary figures and images for: EnrichedHeatmap: an R/Bioconductor package for comprehensive visualization of genomic signal associations
Source: BMC Genomics. 2018 Apr 4;19:234. doi: 10.1186/s12864-018-4625-x (PMC5885322; doi:10.1186/s12864-018-4625-x)

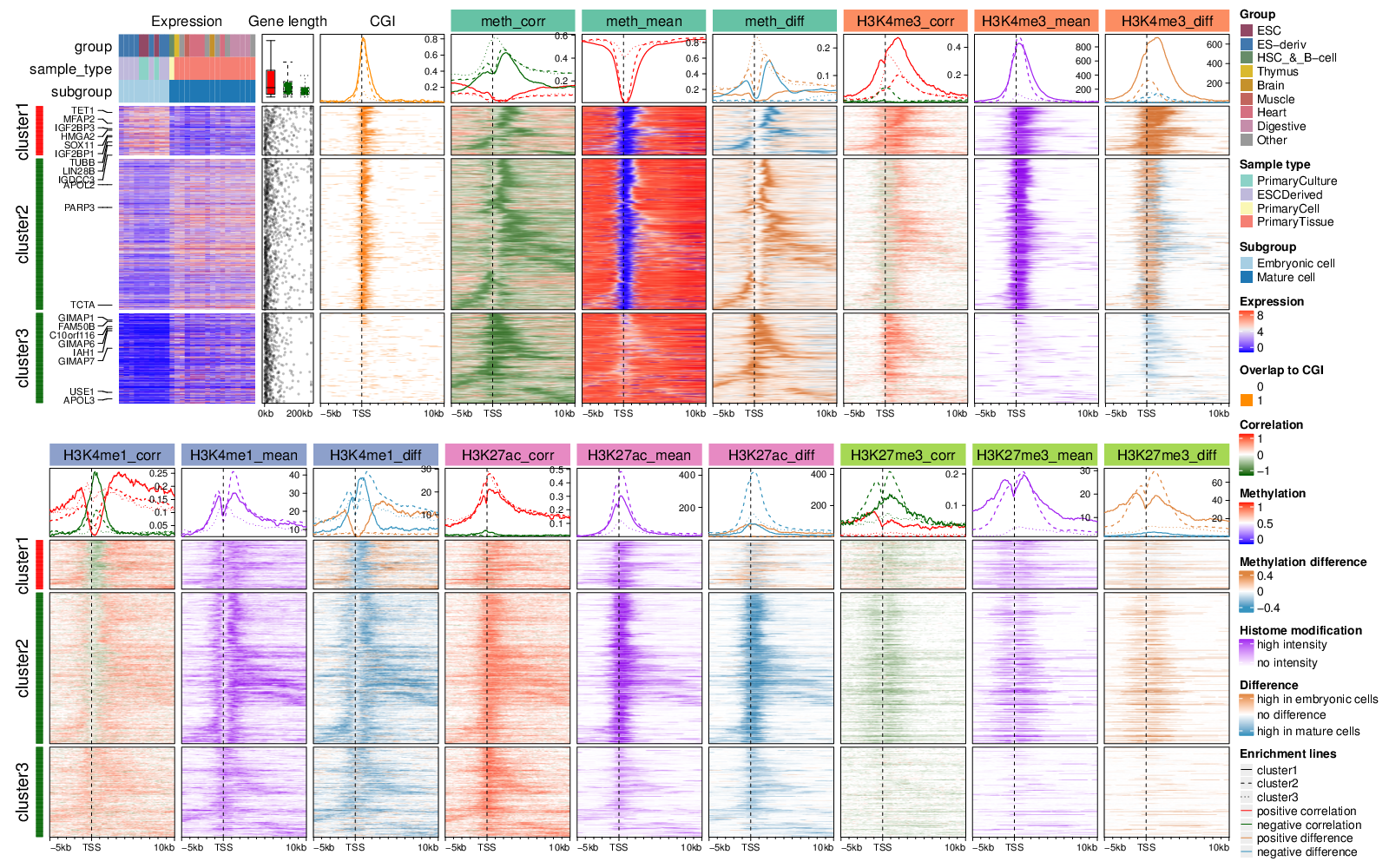

Supplement: Supplementary file 1 — Data and source code for producing Figs. 1 and 2. (GZ 45195 kb) [file 12864_2018_4625_MOESM1_ESM.gz › EnrichedHeatmap-supplementary/roadmap.png]

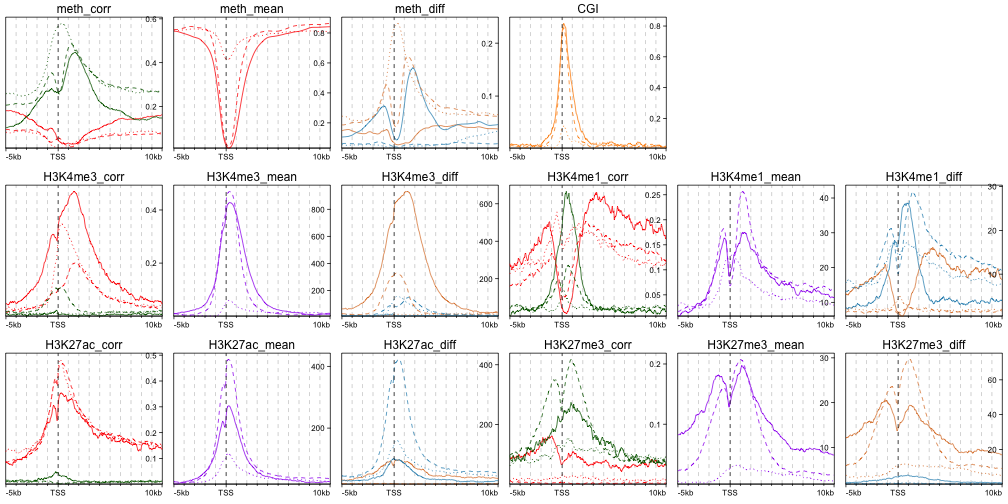

Supplement: Supplementary file 1 — Data and source code for producing Figs. 1 and 2. (GZ 45195 kb) [file 12864_2018_4625_MOESM1_ESM.gz › EnrichedHeatmap-supplementary/figure/unnamed-chunk-30-1.png]

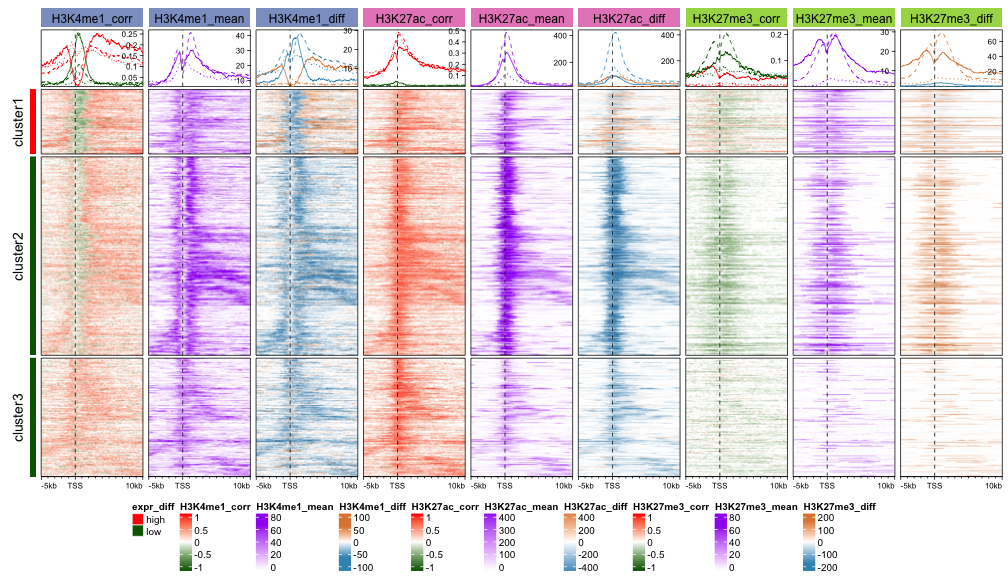

Supplement: Supplementary file 1 — Data and source code for producing Figs. 1 and 2. (GZ 45195 kb) [file 12864_2018_4625_MOESM1_ESM.gz › EnrichedHeatmap-supplementary/figure/unnamed-chunk-29-1.png]

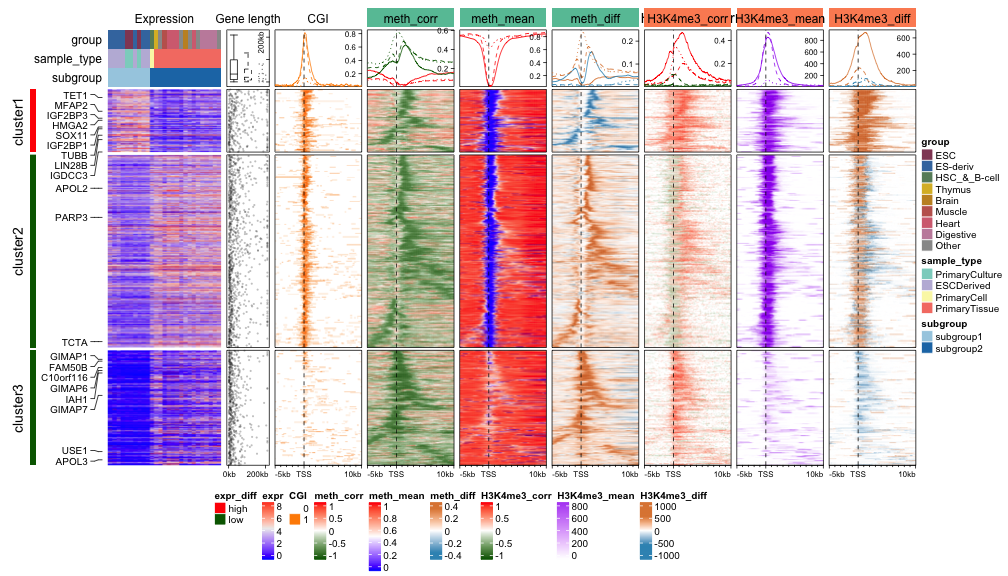

Supplement: Supplementary file 1 — Data and source code for producing Figs. 1 and 2. (GZ 45195 kb) [file 12864_2018_4625_MOESM1_ESM.gz › EnrichedHeatmap-supplementary/figure/unnamed-chunk-28-1.png]

A

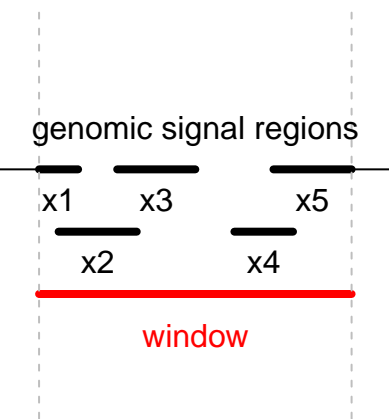

B

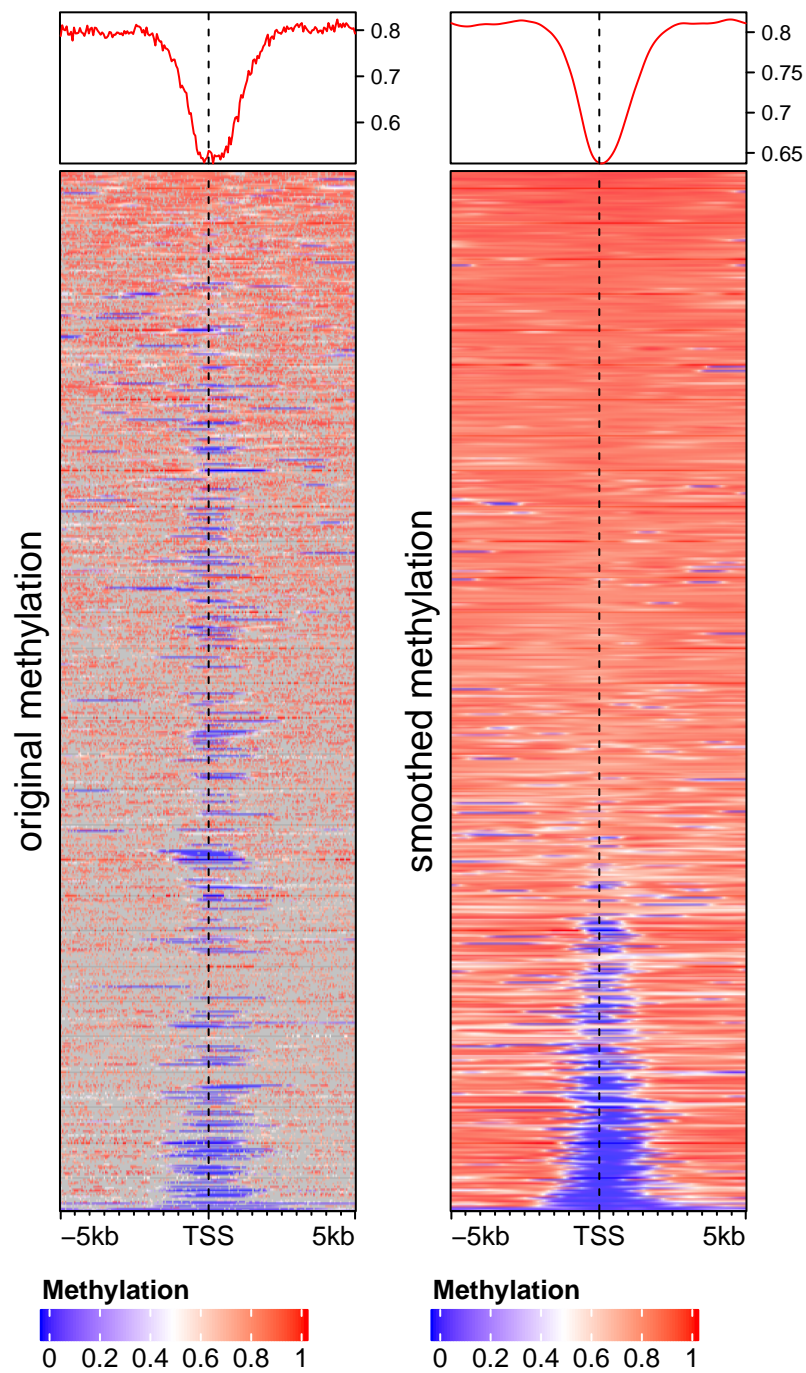

C

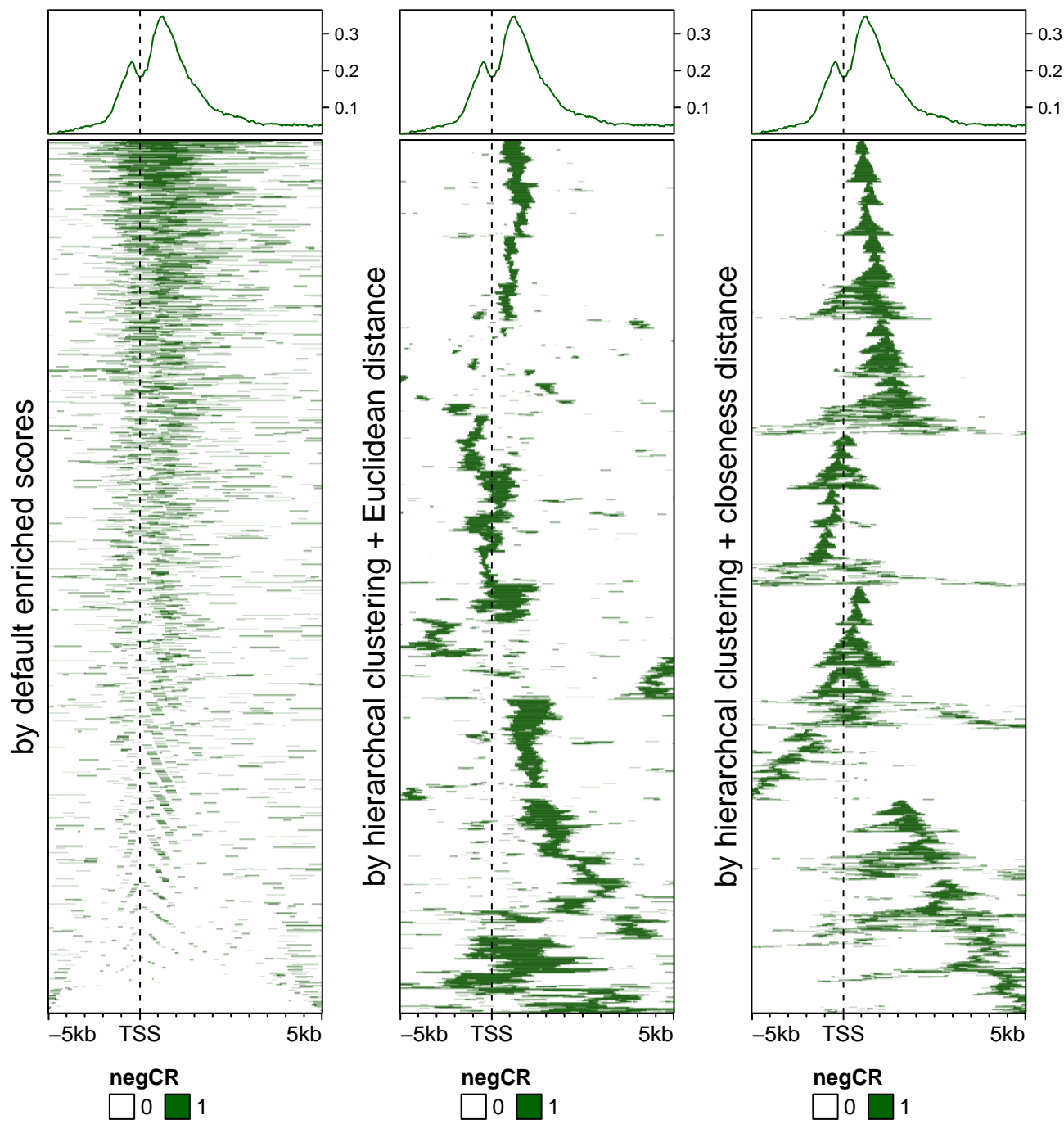

Supplement: Supplementary file 1 — Data and source code for producing Figs. 1 and 2. (GZ 45195 kb) [file 12864_2018_4625_MOESM1_ESM.gz › EnrichedHeatmap-supplementary/figure1.pdf]

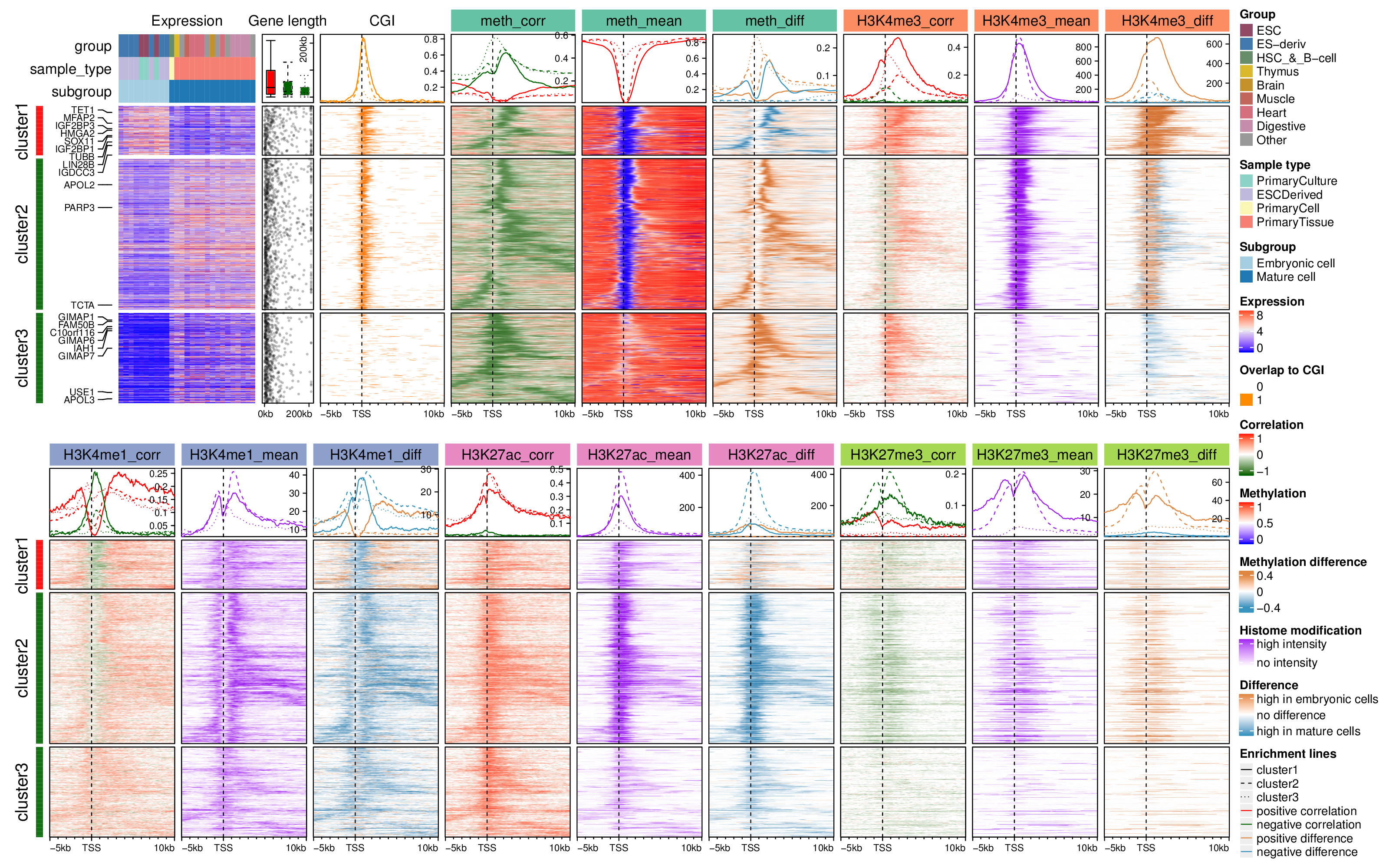

Supplement: Supplementary file 1 — Data and source code for producing Figs. 1 and 2. (GZ 45195 kb) [file 12864_2018_4625_MOESM1_ESM.gz › EnrichedHeatmap-supplementary/figure2.png]

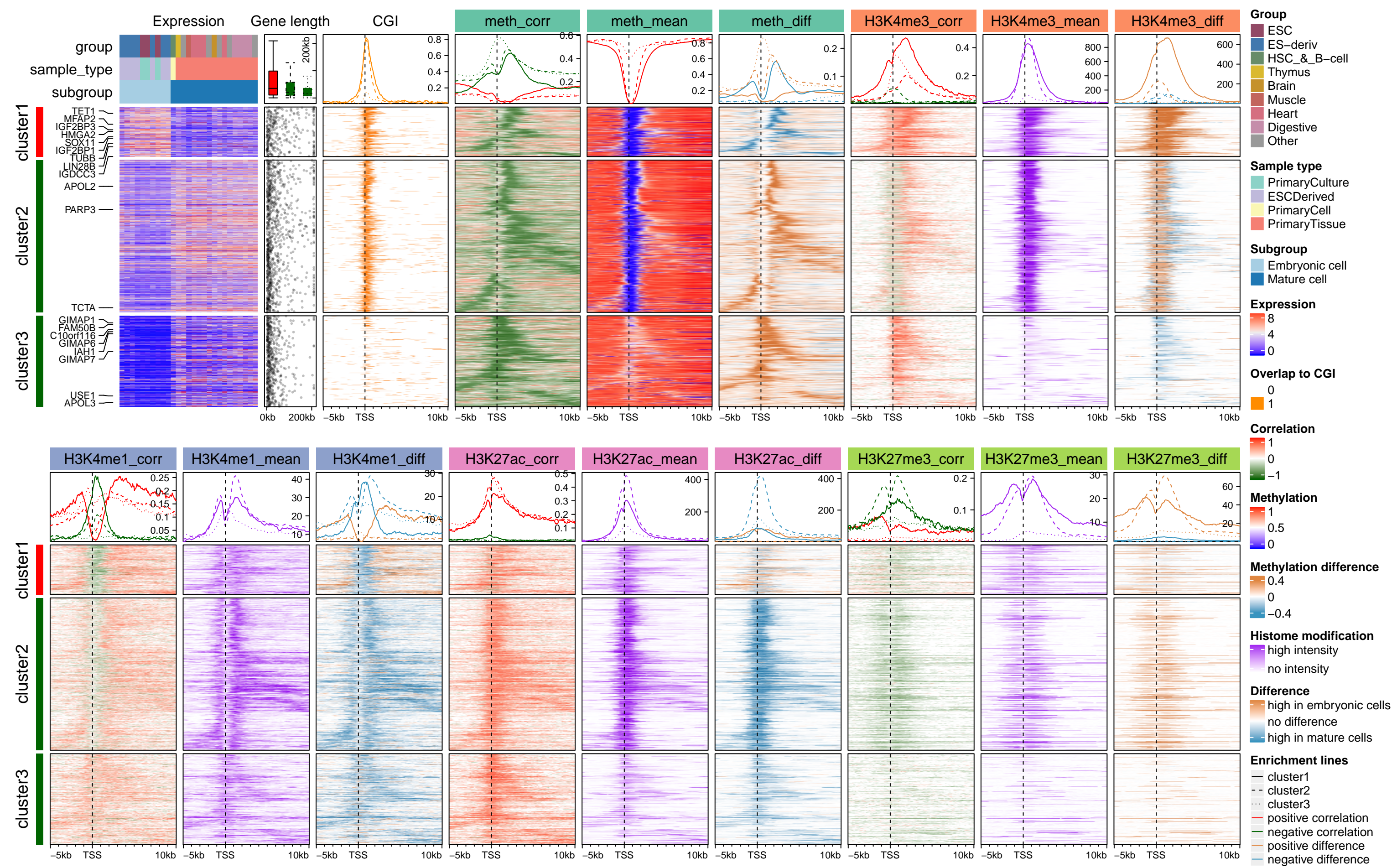

Supplement: Supplementary file 1 — Data and source code for producing Figs. 1 and 2. (GZ 45195 kb) [file 12864_2018_4625_MOESM1_ESM.gz › EnrichedHeatmap-supplementary/figure2.pdf]
